# Focused Examination of the Intestinal Epithelium Reveals Transcriptional Signatures Consistent with Disturbances in Enterocyte Maturation and Differentiation during the Course of SIV Infection

**DOI:** 10.1371/journal.pone.0060122

**Published:** 2013-04-09

**Authors:** Mahesh Mohan, Deepak Kaushal, Pyone P. Aye, Xavier Alvarez, Ronald S. Veazey, Andrew A. Lackner

**Affiliations:** 1 Division of Comparative Pathology, Tulane National Primate Research Center, Covington, Louisiana, United States of America; 2 Division of Bacteriology and Parasitology, Tulane National Primate Research Center, Covington, Louisiana, United States of America; Commissariat a l'Energie Atomique(cea), France

## Abstract

The Gastrointestinal (GI) tract plays a pivotal role in AIDS pathogenesis as it is the primary site for viral transmission, replication and CD4^+^ T cell destruction. Accordingly, GI disease (enteropathy) has become a well-known complication and a driver of AIDS progression. To better understand the molecular mechanisms underlying GI disease we analyzed global gene expression profiles sequentially in the intestinal epithelium of the same animals before SIV infection and at 21 and 90 days post infection (DPI). More importantly we obtained sequential excisional intestinal biopsies and examined distinct mucosal components (epithelium. intraepithelial lymphocytes, lamina propria lymphocytes, fibrovascular stroma) separately. Here we report data pertaining to the epithelium. Overall genes associated with epithelial cell renewal/proliferation/differentiation, permeability and adhesion were significantly down regulated (<1.5–7 fold) at 21 and 90DPI. Genes regulating focal adhesions (n = 6), gap junctions (n = 3), ErbB (n = 3) and Wnt signaling (n = 4) were markedly down at 21DPI and the number of genes in each of these groups that were down regulated doubled between 21 and 90DPI. Notable genes included FAK, ITGA6, PDGF, TGFβ3, Ezrin, FZD6, WNT10A, and TCF7L2. In addition, at 90DPI genes regulating ECM-receptor interactions (laminins and ITGB1), epithelial cell gene expression (PDX1, KLF6), polarity/tight junction formation (PARD3B&6B) and histone demethylase (JMJD3) were also down regulated. In contrast, expression of NOTCH3, notch target genes (HES4, HES7) and EZH2 (histone methyltransferase) were significantly increased at 90DPI. The altered expression of genes linked to Wnt signaling together with decreased expression of PDX1, PARD3B, PARD6B and SDK1 suggests marked perturbations in intestinal epithelial function and homeostasis leading to breakdown of the mucosal barrier. More importantly, the divergent expression patterns of *EZH2* and *JMJD3* suggests that an epigenetic mechanism involving histone modifications may contribute to the massive decrease in gene expression at 90DPI leading to defects in enterocyte maturation and differentiation.

## Introduction

HIV/SIV infection of the gastrointestinal (GI) tract results in massive destruction of CD4^+^ T cells, increased viral replication and persistent inflammation resulting in significant damage to GI structure and function [1]–[6]. The damage inflicted to the GI tract both directly by the virus and indirectly by the host's immune/inflammatory response generally involves all mucosal compartments (epithelium, lamina propria cells, fibrovascular stroma., etc) and plays an important role in driving AIDS progression [7]–[10]. Consequently, comprehending the underlying molecular mechanisms/pathology will require a detailed dissection of the molecular pathological changes occurring in each of these mucosal compartments. Despite the widespread attention this area of research has received in recent years the approaches taken by the majority of published studies have involved the use of intact intestinal segments or pinch endoscopic biopsies. A major shortcoming with these approaches is the difficulty to assign a particular transcriptional signature, be it normal or pathological, conclusively to a certain cellular/mucosal compartment. Further, in HIV/SIV infection the dramatic shifts in lymphocyte populations particularly in the lamina propria in response to viral replication can significantly mask molecular pathological events evolving in other mucosal compartments, most notably, the intestinal epithelium [1]. Furthermore, certain expression signatures from one mucosal compartment (e.g. epithelium) can mask similar but opposite trending expression profiles from another compartment (e. g. lamina propria) leading to inadvertent loss of valuable information [11]. To circumvent these problems we have utilized a novel strategy to minimize the complexity of the intestinal tissue so that information gathering can be maximized [12]. As part of this strategy, we separated intact intestinal segments into distinct mucosal compartments, namely, epithelium, intraepithelial lymphocytes, lamina propria leukocytes and fibrovascular stroma. Additionally, this strategy also involved the comparison of gene expression profiles in intestinal resection segments (∼6–8 cm) obtained from the same animal before and at, at least, two different time points after SIV infection, thus, minimizing animal to animal variation [12].

Employing this novel strategy we recently reported gene expression profiles in intestinal lamina propria leukocytes (LPLs) at 21 and 90DPI. In general our findings were in agreement with previous studies showing that during acute and chronic SIV infection, generalized T-cell activation is accompanied by B-cell and macrophage dysfunction, T-cell apoptosis, dysregulated antiviral signaling and microbial translocation [12]. But more importantly we identified several new transcriptional signatures involved in each of the pathological processes mentioned above. Most notable was massive down-regulation of oxidative phosphorylation genes (n = 50) at 21DPI, a molecular signature indirectly suggesting T cell activation [12].

The intestinal epithelium plays a critical role in maintaining mucosal immune homeostasis. Whereas the intestinal immune system, in general, has been the prime focus of investigation in HIV/SIV research, there has been little attention focused on the intestinal epithelium. Maintaining a healthy and intact intestinal epithelium is critical for barrier function, water/nutrient absorption, antimicrobial immune response and absorption and uptake of orally administered anti-retroviral medication. Further, dysregulated epithelial barrier function can trigger and perpetuate inflammation, allow the influx of intestinal bacteria and their products (often referred to as microbial translocation) and contribute to chronic immune activation and AIDS progression [10]. While recent studies have drawn a link between defects in intestinal epithelial permeability and HIV disease progression [10], a detailed longitudinal examination of the molecular pathological changes taking place exclusively in the intestinal epithelium during the course of HIV/SIV infection is lacking. Using this unique approach in the present study we report gene expression profiles in the intestinal epithelium at 21 and 90DPI. Our results suggest marked dysregulation in the expression of cell-cell and cell-matrix adhesion molecules including critical signaling molecules belonging to the apoptosis, *Wnt-TCF7L2*, *Ephrin* and *Notch* signaling pathways. Overall the transcriptional signatures uncovered from the present study point toward mounting disturbances in enterocyte maturation and differentiation which could contribute to dysfunction of intestinal barrier function and microbial translocation which has been proposed as a driver of AIDS disease progression.

## Results

### Viral load and loss of mucosal CD4+ T cells

**Figure 1:**
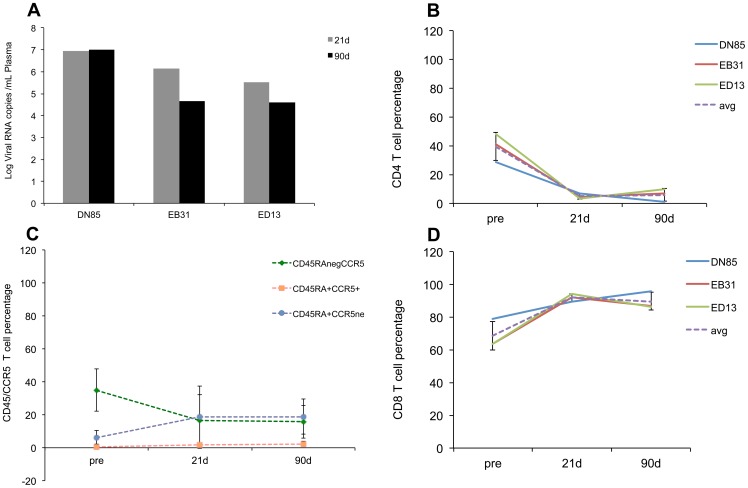
SIV infection results in elevated plasma viremia and rapid depletion of mucosal CD4+ T cells. Plasma viral loads (A) and changes in mucosal CD4+ T cells (B) “memory” CD4^+^ T cells (CD45RAneg, CCR5+) (C), and mucosal CD8^+^ T cells (D) in three Indian origin rhesus macaques at 21 and 90 days after intravenous infection with SIVmac251. Changes in CD45RA+/CCR5- populations at the 21 and 90d timepoints were not statistically significant (p>0.05).

Infection of rhesus macaques with SIV results in high acute plasma viral loads and rapid loss of mucosal CD4^+^ T cells [2]–[6]. Consistent with these prior observations the animals used in this study had high plasma (Fig 1A) and tissue (Fig S1) viral loads and a rapid and profound loss of intestinal CD4^+^ T cells with a nadir at 21DPI (Fig 1B). Figure 1C further shows that the loss of CD4^+^ T lymphocytes was primarily due to loss of the “memory” population that is CD45RA negative and CCR5 positive. The loss of mucosal CD4^+^ T cells was accompanied by a concomitant increase in CD8^+^ T cells (Fig 1D) at 21 and 90DPI. Intestinal tissues were histologically examined by a board certified pathologist. No significant histopathologic abnormalities (inflammation, villus blunting, etc) were observed.

### Gene expression profiles during acute SIV infection in the jejunal epithelium are indicative of early signaling events associated with epithelial cell loss and increased cellular proliferation to restore epithelial integrity

To determine the impact of viral replication in the intestinal lamina propria on the epithelial cell transcriptome we performed genome wide gene expression profiling focused on jejunal epithelial cells obtained from the same animals before and at 21 and 90DPI. Differentially expressed transcripts selected for further analysis were restricted to those whose expression either increased or decreased by, at least, 1.5- fold (*P≤0.05*) in response to SIV infection. Trypan blue staining revealed that more than 90% of the epithelial cell suspensions isolated from all three animals were viable. With the exception of three genes specific for CD8 T cells or NK cells the vast majority of the genes were epithelium specific suggesting minimal contamination of other cell types. The immune cell specific genes detected were: 1) T-cell surface glycoprotein CD8 beta chain precursor (CD8b antigen) at 21 days PI; 2) Natural killer cell receptor 2B4 precursor (NK cell type I receptor protein 2B4; CD244 antigen) at 90 days PI; and 3) Natural killer-tumor recognition sequence isoform a.

Following analysis using DAVID [13]–[14] and GeneCards [15] we found 525 distinct transcripts to be up-regulated in the epithelium at 21DPI (Table 1). A modestly increased number of transcripts showed decreased expression at this time point (n = 655). In contrast, at 90DPI the number of transcripts with decreased expression (n = 1605) was more than 3 times greater than the up-regulated transcripts (n = 526) (p = 0.0001) and nearly 2.5 times greater than were down-regulated at 21DPI (n = 655) (p = 0.0001). This data illustrates that the transcription profile is one of progressive down-regulation of intestinal epithelial genes. Tables 2 and 3 provide information on fold difference and *p* values for a select number of differentially expressed (up and down) transcripts at 21 and 90DPI. The entire list of differentially expressed genes in acute (21DPI) and chronic (90DPI) infection with their affymetrix IDs, *p* values and fold difference is provided in Table S1 and Table S2.

**Table 1:** Differentially expressed genes in the intestinal epithelium during acute (21DPI) and chronic (90DPI) SIV infection.

	Total genes	Known genes	Unknown genes	Annotated genes
**Up regulated**				
21 D PI	525	426	99	426
90 D PI	526	420	106	420
**Down regulated**				
21 D PI	655	533	122	533
90 D PI	1605	1353	252	1353

**Table 2:** Select list of differentially expressed genes in the Intestinal Epithelium at 21 days post SIV infection.

Gene ID	Symbol	Fold Difference	P value
**Up vs Preinfection**			
CASP2 and RIPK1 domain containing adaptor with death domain	*CRADD*	1.6	0.017
Forkhead box O3	*FOXO3*	1.6	0.036
Forkhead box O1	*FOXO1*	1.9	0.024
Serine/threonine kinase 17a	*STK17A*	2.2	3E-05
Serine/theronine kinase 3	*STK3*	2	0.039
Tumor necrosis factor receptor superfamily, member 25	*TNFRSF25*	4.4	0.007
WT1-interacting protein		2.6	0.003
Protein phosphatase 1f	*PPM1F*	1.5	0.037
Forkhead transcription factor A2	*FOXA2*	2	0.032
Runt-related transcription factor 1	*RUNX1*	4.1	0.019
Enhancer of zeste homolog 1	*EZH1*	2.1	0.049
Kruppel-like factor 7	*KLF7*	2.3	0.016
RAR-related orphan receptor A	*RORA*	3.6	0.046
beta-defensin 1	*DEFB1*	1.6	1E-04
Toll-like receptor 9	*TLR9*	1.8	0.028
Intestinal mucin 3A	*MUC3A*	3.8	0.018
Interleukin 12B	*IL12B*	3	0.046
Cyclin D3	*CCND3*	1.7	0.007
Hairy and enhancer of split 6	*HES6*	1.6	0.038
EPH receptor A2	*EPHA2*	1.7	0.043
EPH receptor B3 precursor	*EPHB3*	1.7	0.023
Bone morphogenetic protein 6	*BMP6*	1.9	0.02
Delta-like 4 (Drosophila)	*DLL4*	1.6	0.022
SOCS1	*SOCS1*	1.7	0.026
Integrin alpha L	*ITGAL*	2.9	0.011
Cell adhesion molecule JCAM	*JCAM*	2.4	0.018
Laminin, alpha 1	*LAMA1*	1.9	0.039
Thrombospondin 1	*THBS1*	2.5	0.001
Mucin 1	*MUC1*	2.5	0.016
Dishevelled 2	*DVL2*	1.8	0.025
Mitogen-activated protein kinase kinase kinase 2	*MAP3K2*	1.6	0.032
**Down vs Preinfection**			
Pancreatic and duodenal homeobox 1	*PDX1*	3.5	0.01
Transcription factor 7-like 2	*TCF7L2*	3.1	0.045
Homeobox protein Hox-D8	*HOXD8*	1.9	0.025
Mastermind-like 3	*MAML3*	2.5	0.01
SRY (sex determining region Y)-box 2	*SOX2*	3	0.003
Integrin alpha 6	*ITGA6*	2.5	0.023
Integrin beta 1	*ITGB1*	2.9	0.014
PTK2 protein tyrosine kinase 2	*PTK2*	1.8	0.029
V-set and immunoglobulin domain containing 1	*VSIG1*	1.9	0.019
Cadherin 5	*CDH5*	2.1	0.045
Catenin (cadherin-associated protein), alpha 3	*CTNNA3*	3	0.003
Claudin 22	*CLDN22*	2.9	0.029
Frizzled homolog 6 (Drosophila)	*FZD6*	2.1	0.039
Wingless-type MMTV integration site family, member 10A	*WNT10A*	1.9	0.026
TGF beta receptor associated protein −1		1.5	0.039
Ephrin-A2 precursor	*EPHA8*	1.6	0.033
Transforming growth factor beta 3	*TGFB3*	1.9	0.044
Cadherin 5	*CDH5*	2.1	0.045
Catenin (cadherin-associated protein), alpha 3	*CTNNA3*	3	0.003
Claudin 22	*CLDN22*	2.9	0.029
Contactin 1	*CNTN1*	2	0.041
**Down vs 90 DPI**			
GATA6	*GATA6*	2.6	0.019
Caudal type homeobox 2	*CDX2*	2	0.011
Intestinal alkaline phosphatase	*ALP1*	4.9	0.011
Zinc finger and BTB domain containing 33	*ZBTB33*	2.2	0.045
Inversin	*INVS*	2.4	0.047
Leucine rich repeat (in FLII) interacting protein 2	*LRRFIP2*	2	0.04
low density lipoprotein receptor-related protein 5	*LRP5*	2.4	0.026

**Table 3:** Select list of differentially expressed genes in the Intestinal Epithelium at 90 days post SIV infection.

Gene ID	Symbol	Fold Difference	P value
**Up vs Preinfection**			
Cyclin-dependent kinase inhibitor 2D (p19, inhibits CDK4)	*CDKN2D*	3.21	0.027
Fibroblast growth factor 4	*FGF4*	2.64	0.01
Platelet derived growth factor D	*PDGFD*	3.04	0.022
Ras homolog gene family, member H	*RHOH*	2.09	0.028
Inscuteable homolog (Drosophila)	*INSC*	3.23	0.004
Kruppel-like factor 12	*KLF12*	4.38	0.038
NK2 homeobox 2	*NKX2-2*	1.62	0.006
Enhancer of zeste homolog 2 (Drosophila)	*EZH2*	1.69	0.02
Ets homologous factor	*EHF*	2.02	0.021
Hairy and enhancer of split 4 (Drosophila)	*HES4*	3.73	0.04
Runt-related transcription factor 1	*RUNX1*	3.82	0.042
Hairy and enhancer of split 7	*HES7*	2.34	0.046
Defensin, beta 119	*DEFB119*	2	0.025
Mucin 5B, oligomeric mucus/gel-forming	*MUC5B*	1.81	0.01
T cell immunoglobulin mucin 3	*HAVCR2*	2.45	0.031
Janus kinase 1	*JAK1*	2.49	0.006
Notch homolog 3 (Drosophila)	*NOTCH3*	2.08	0.045
Bone morphogenetic protein receptor, type IA	*BMPR1A*	2.03	0.039
Dickkopf homolog 3	*DKK3*	3.35	0.042
Inositol polyphosphate-4-phosphatase, type I, 107kDa	*INPP4A*	1.66	0.022
kringle containing transmembrane protein 1	*KREMEN1*	2.31	0.049
Deltex 3 homolog	*DTX3*	2.75	0.002
Suppressor of cytokine signaling 1	*SOCS1*	1.53	0.017
Wingless-type MMTV integration site family, member 7B	*WNT7B*	2.46	0.038
Deltex homolog 1	*DTX1*	2.35	0.044
Cadherin 8, type 2	*CDH8*	2.22	0.01
Dishevelled associated activator of morphogenesis 1	*DAAM1*	3.34	0.045
**Down vs Preinfection**			
Paraoxonase 2	*PON2*	2.3	0.031
Dishevelled, dsh homolog 1 (Drosophila)	*DVL1*	1.9	0.038
Pancreatic and duodenal homeobox 1	*PDX1*	3.2	0.017
Transcription factor 7-like 2	*TCF7L2*	3.1	0.017
PTK2 protein tyrosine kinase 2	*PTK2*	2.4	0.045
Frizzled-related protein	*FRZB*	3.3	0.032
Mucin 13, epithelial transmembrane	*MUC13*	7.3	0.034
Dickkopf homolog 3	*DKK3*	2.5	0.034
Cadherin 5, type 2	*CDH5*	2.7	0.026
Catenin (cadherin-associated protein), alpha 1, 102kDa	*CTNNA1*	2.1	0.024
Integrin, alpha 6	*ITGA6*	2.7	0.008
Laminin, gamma 2	*LAMC2*	2.4	0.04
Par-3 partitioning defective 3 homolog	*PARD3*	6.9	0.013
Par-6 partitioning defective 6 homolog gamma	*PARD6G*	4.3	0.012
Desmocollin 2	*DSC2*	2.1	0.036
Junction plakoglobin	*JUP*	2.7	0.021
Villin 2	*VIL2*	3.9	0.001
**Up vs 21 DPI**			
Runt related transcription factor 2	*RUNX2*	1.5	0.031
Sry homeobox 2	*SRYX2*	2.7	0.042
Nucleotide oligomerization domain containing 1	*NOD1*	1.5	0.035
Dickkopf homolog 3	*DKK3*	3.5	0.003
Ring finger protein 138	*RNF138*	2.2	0.03
EPH receptor A6	*EPHA6*	2.2	0.005
Ubiquitin specific peptidase 34	*USP34*	1.5	0.037

Approximately 426 out of 525 up-regulated genes were found to be annotated at 21DPI (Table 1). Table 2 shows fold difference and *p* values for select transcripts important to HIV/SIV infection. With the help of both tools we sorted all differentially expressed genes into 10 different categories, namely; transcription, immune defense/inflammation, cell division/differentiation, cell signaling, cell adhesion/migration, transport, DNA replication/repair, regulation of cellular cytoskeleton, apoptosis, metabolism and transcripts with unknown function.

**Figure 2:**
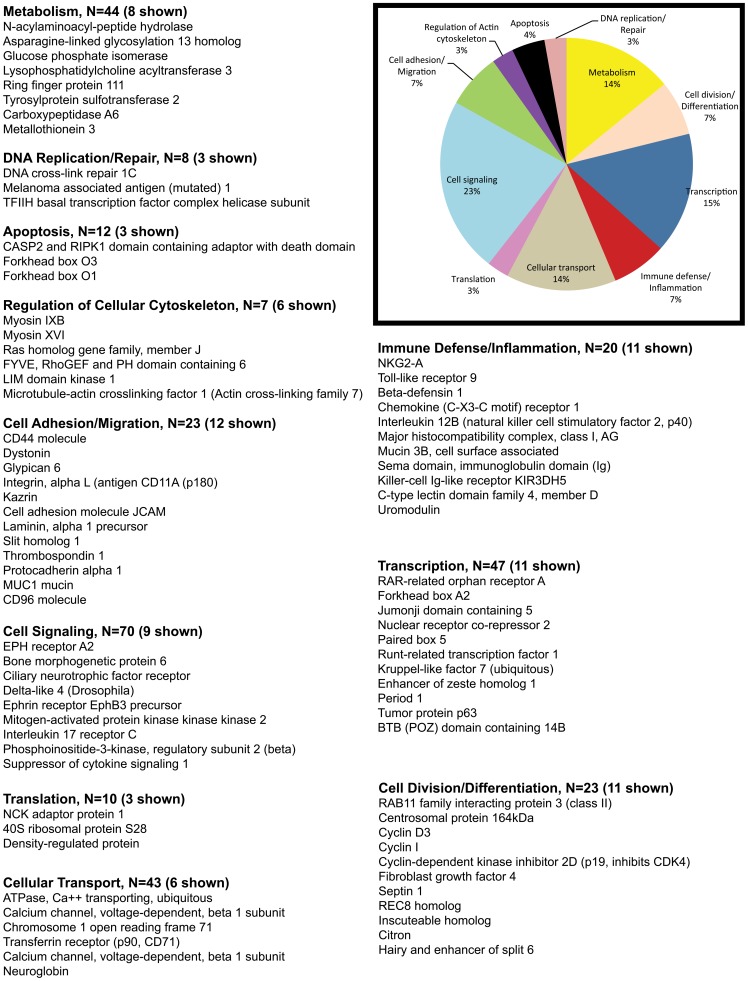
Gene functional categories up (1.5 fold) in the intestinal epithelium at 21DPI. The relative size of each sector in the pie chart is determined by the number of genes in that functional category. Genes with unknown function are not included in the pie chart. Only a few transcripts of importance to SIV infection are shown in the figure under each functional category. The full list of genes grouped under each functional category for the 21DPI time point is provided in supplementary table S1.

**Figure 3:**
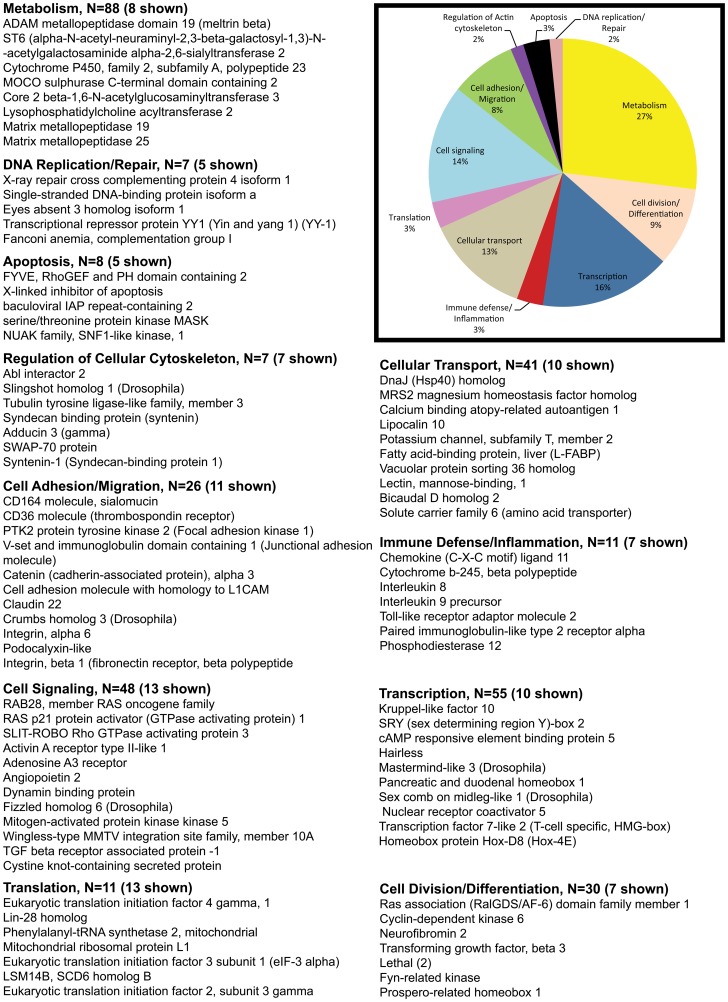
Gene functional categories down (1.5 fold) in the intestinal epithelium at 21DPI. The relative size of each sector in the pie chart is determined by the number of genes in that functional category. Genes with unknown function are not included in the pie chart. Only a few transcripts of importance to SIV infection are shown in the figure under each functional category. The full list of genes grouped under each functional category for the 21DPI time point is provided in supplementary table S1.

**Figure 4:**
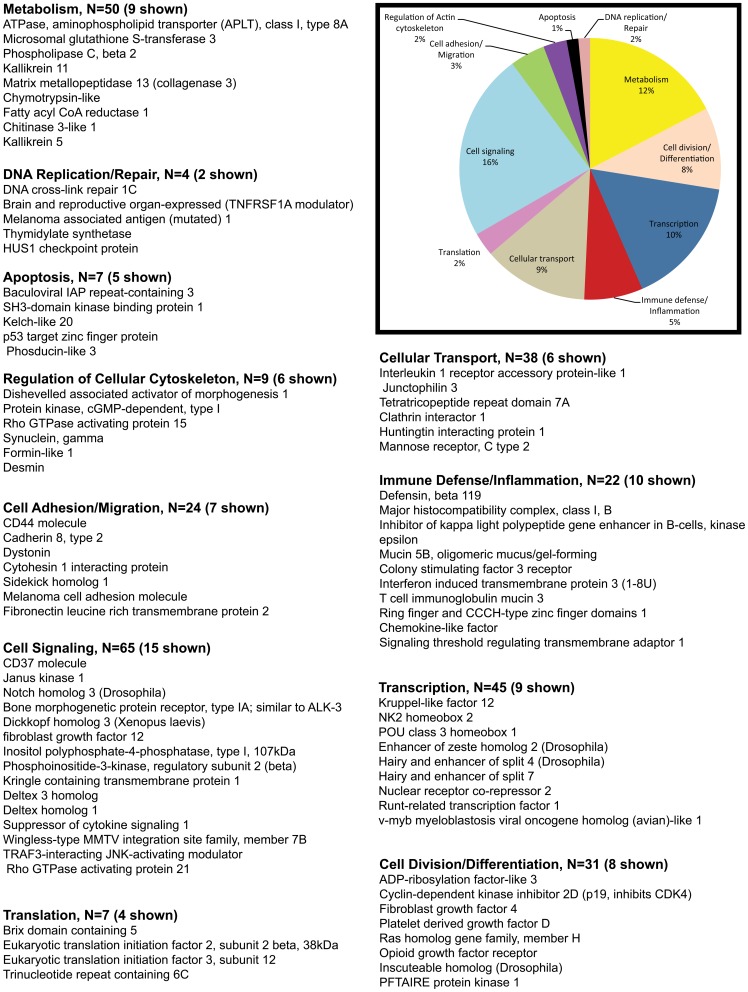
Gene functional categories up (1.5 fold) in the intestinal epithelium at 90DPI. The relative size of each sector in the pie chart is determined by the number of genes in that functional category. Genes with unknown function are not included in the pie chart. Only a few transcripts of importance to SIV infection are shown in the figure under each functional category. The full list of genes grouped under each functional category for the 90d time point is provided in supplementary table S2.

**Figure 5:**
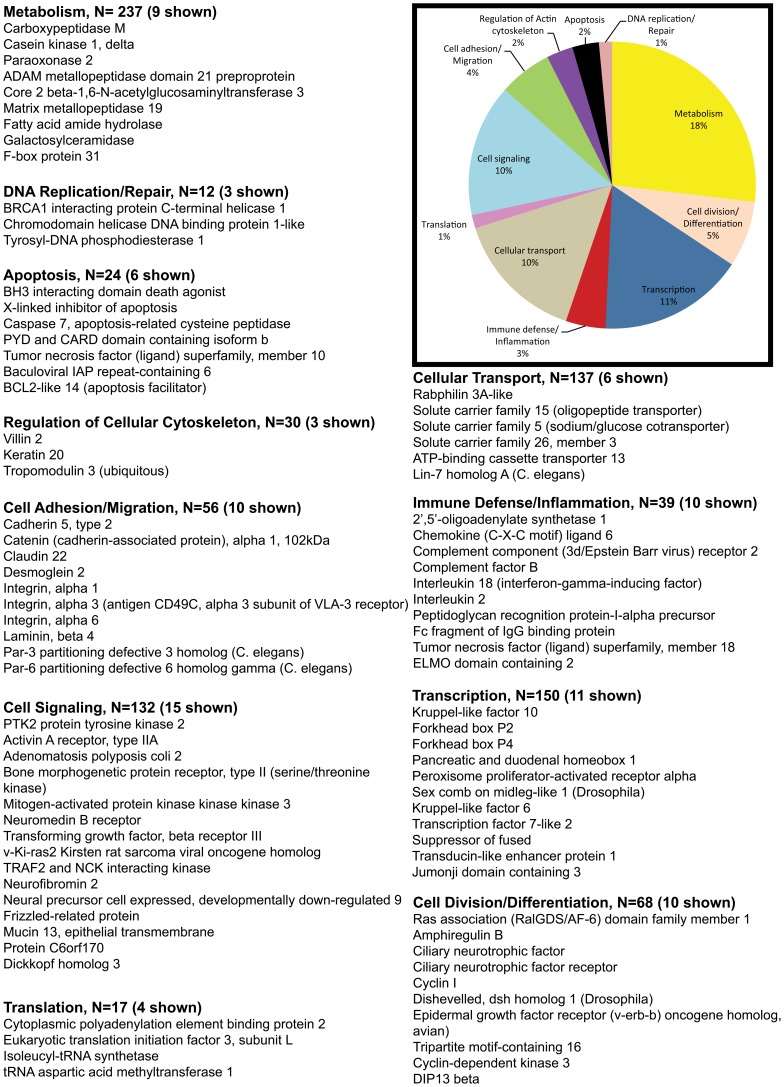
Gene functional categories down (1.5 fold) in the intestinal epithelium at 90DPI. The relative size of each sector in the pie chart is determined by the number of genes in that functional category. Genes with unknown function are not included in the pie chart. Only a few transcripts of importance to SIV infection are shown in the figure under each functional category. The full list of genes grouped under each functional category for the 90d time point is provided in supplementary table S2.

The pie charts (Figures 2, 3, 4, 5) show the percentage of the total number of differentially expressed genes that fell in the 11 functional annotation categories (metabolism, inflammation/immune defense etc.) common to all three animals at 21 and 90DPI time points. The unknown transcripts are not represented in the pie charts shown in Figures 2, 3, 4, 5.

Based on gene ontology/annotation, the 21DPI time point (the nadir of CD4^+^ T cell loss) yielded valuable information on the early pathological events occurring in the intestinal epithelium following SIV infection (Fig 2). A major finding at this time point was a significant increase in the expression of pro-apoptotic genes (∼4% n = 12). Some important apoptosis related genes that fell in this category included *CASP2 and RIPK1 domain containing adaptor with death domain, forkhead box O3 (FOXO3), FOXO1, serine/threonine kinase 17a, serine/theronine kinase 3, tumor necrosis factor receptor superfamily, member 25, WT1-interacting protein, and protein phosphatase 1f.*

Genes associated with cellular transcription accounted for ∼15% (n = 47) of differentially expressed genes (Fig 2). Differentially expressed genes known to regulate transcription included *FOXA2 (enteroendocrine and goblet cell differentiation), RUNX1 (goblet cell differentiation), jumonji domain containing 5 (JDC5) (histone methylase), enhancer of zeste homolog 1 (histone methyltransferase), Kruppel-like factor 7 (KLF7), nuclear receptor co-repressor 2 (Co-repressor), group A, and RAR-related orphan receptor A) (coactivator)*.

Genes associated with immune defense/inflammation accounted for ∼7% (n = 20) of the up-regulated genes (Fig 2). Prominent members in the immune defense/inflammation category included *beta-defensin 1 (antimicrobial), chemokine (C-X3-C motif) receptor 1, toll-like receptor 9 (TLR9), intestinal mucin 3A (antimicrobial), interleukin 12B (IL-12B) (pro-inflammatory)*.

Notable genes regulating cell division/differentiation (7%, n = 23) included *cyclin I, cyclin D3, fibroblast growth factor 4 (FGF4), hairy and enhancer of split 6 [HES6] (cell differentiation), inscuteable homolog, and cyclin-dependent kinase inhibitor 2D (p19, inhibits CDK4)*.

Approximately, 23% of the transcripts included cell signaling genes (n = 70) such as *Ephrin (EPH) receptor A2, EPH receptor B3, bone morphogenetic protein 6, ciliary neurotrophic factor receptor, interleukin 2 receptor, beta, delta-like 4 (Drosophila), opioid receptor, mu 1, dishevelled 2, mitogen-activated protein kinase kinase kinase 2, mitogen-activated protein kinase kinase 7, TRAF3-interacting JNK-activating modulator, interleukin 17 receptor C and SOCS1 (negative regulator of JAK-STAT pathway)*.

Another 7% included genes associated with cell adhesion/migration (n = 23) that included *CD44, dystonin, endothelial cell adhesion molecule, integrin alpha L (leukocyte recruitment/trafficking), kazrin, cell adhesion molecule JCAM, laminin, alpha 1, thrombospondin 1, protocadherin alpha 1, mucin 1 (MUC1), and CD96 molecule*.

About 3% (n = 7) of the transcripts represented genes regulating cellular cytoskeleton (*gelsolin, moesin, TBC1 domain family member 10A, actinin, and alpha 4 smooth muscle myosin heavy chain 11 isoform SM1A*). The remaining transcripts fell into the following functional categories: metabolism (14%, n = 44), cellular transport (14%, n = 43), DNA replication/repair (3%, n = 8), and translation (3%, n = 10) (Fig 2).

Collectively, the transcriptional profile at 21DPI provides clear evidence of increased intestinal epithelial cell apoptosis and a substantial effort to proliferate (*cyclin 1, D3*), migrate (*EPH ligand and receptor*) and differentiate (*FOXA2, HES6*) into mature enterocytes so that some of the key functions of the intestine such as nutrient absorption, barrier function and antimicrobial functions (*beta defensin 1, intestinal mucin 3A*) are maintained or restored.

### Decreased expression of genes encoding apoptosis inhibitors, focal adhesion kinase, and key cell adhesion molecules in the intestinal epithelium at 21DPI although consistent with the early enterocyte loss also suggests aberrations in mucosal repair and healing resulting in incomplete restoration of epithelial integrity

Compared to the upregulated gene list (n = 525) about 20% more (n = 655) genes were down-regulated at 21DPI (Table 1). About 533 of these down-regulated genes were found to be annotated (Table 1). The exact reason/s for this considerable decrease in gene expression are unclear but could be attributable to the massive apoptosis of intestinal epithelial cells reported previously [16]–[17] and correlates well with the increased expression of pro-apoptotic genes we observed in the present study (Fig 2). More importantly, several of the down-regulated transcripts were represented by genes linked to the canonical Wnt-TCF7L2, TGFβ signaling pathway, cell-cell, cell-matrix adhesion and inhibition of apoptosis.

Similar to the up-regulated group, the percentage of down-regulated genes controlling transcription (16%, n = 55), cell adhesion/migration (8%, n = 26), cell division/differentiation (9%, n = 30), cellular transport (13%, n = 41), translation (3%, n = 11), regulation of cellular cytoskeleton (3%, n = 7), apoptosis (3%, n = 8) and DNA replication/repair (2%, n = 7) were comparable (Fig 2 & 3).

Notable genes regulating transcription in epithelial cells that were decreased included *pancreatic and duodenal homeobox 1 (PDX1), transcription factor 7-like 2 (TCF7L2), homeobox protein Hox-D8 (provides cells with specific positional identities), mastermind-like 3 (Notch signaling partner), sex comb on midleg-like 1 (Transcriptional repression), KLF10, SRY (sex determining region Y)-box 2, cAMP responsive element binding protein 5, and myeloblastosis proto-oncogene*.

Down-regulated genes critical to epithelial cell adhesion included *integrin alpha 6 (ITGA6), integrin beta 1 (fibronectin receptor), CD164 (sialomucin), CD36 molecule (thrombospondin receptor), PTK2 protein tyrosine kinase 2 (focal adhesion kinase 1), v-set and immunoglobulin domain containing 1 (junctional adhesion molecule or JAM), cadherin 5, type 2, catenin (cadherin-associated protein), alpha 3, claudin 22, collagen, type IX, alpha 3, contactin 1, and crumbs homolog 3 (Drosophila)*.

Cell signaling genes down-regulated at the 21DPI timepoint included *activin A receptor type II-like 1, frizzled homolog 6 (Drosophila), WNT10A (wingless-type MMTV integration site family, member 10A), TGF-beta receptor associated protein −1, and Ephrin-A2 precursor*.

Approximately 27% of the down-regulated genes fell under the metabolism category and ∼20% (n = 48) of these were associated with lipid metabolism. Recently, Chen et al [18] reported significant alterations in the expression of genes linked to lipid and iron metabolism in mouse intestinal epithelium following conditional inactivation of *PDX1*. In agreement with these reports, *PDX1* expression was found to be significantly reduced in the present study at 21 and 90DPI and may explain the marked down-regulation of lipid metabolism genes. The decreased expression of *PDX1* may negatively influence enterocyte function as it is known to shape gene expression in enterocytes and enteroendocrine cells [19].

In addition, compared to the 90DPI time point, the 21DPI time point had decreased expression of genes associated with intestinal epithelial cell proliferation and differentiation [*GATA6, caudal type homeobox 2 (CDX2), Jagged 2, intestinal alkaline phosphatase, septin 2, timeless homolog, zinc finger and BTB domain containing 33, early growth response 1, transcription factor AP-2 beta (activating enhancer binding protein 2 beta) and TCF7L2*], transcription [*mastermind-like 2, homeobox B9, zinc finger and SCAN domain containing 10, bromodomain adjacent to zinc finger domain, 2A, and early growth response 4],* and cell signaling *[inversin*, *G protein-coupled receptor, family C, group 5, member A, leucine rich repeat (in FLII) interacting protein 2, low density lipoprotein receptor-related protein 5*. Interestingly, at least, five genes, namely, *zinc finger and BTB domain containing 33* [20], *TCF7L2, inversin* [21], *leucine rich repeat (in FLII) interacting protein 2* [22], *and low density lipoprotein receptor-related protein 5* [23] are linked to the Wnt signaling pathway. The reduced expression of *FAK*, *ITGA6, ITGB1, catenin, PDX1* accompanied by marked perturbations in the expression of genes encoding the different components of the Wnt signaling pathway provides an early indication of impaired epithelial repair/healing and differentiation processes as early as 21DPI.

### Enhanced expression of numerous Wnt and Notch signaling genes at viral set point suggests an effort to maintain progenitor cell proliferation and promote enterocyte differentiation

At viral set point (90DPI) a total of 526 genes were found to be up-regulated (Table 1). Among these 420 were annotated genes (Table 1). Genes regulating cell signaling accounted for 16% (n = 65) of those up-regulated (Fig 4). Table 3 shows fold difference and *p* values for select transcripts important to HIV/SIV infection. Of particular interest were (*CD37, notch homolog 3, bone morphogenetic protein receptor, type IA (cell proliferation), Dickkopf homolog 3, FGF12, deltex homolog 3, deltex homolog 1 (both notch ligands), SOCS1, wingless-type MMTV integration site family, member 7, Rho GTPase activating protein 21B and TRAF3-interacting JNK-activating modulator (transcriptional activator)*.

Genes regulating transcription included *ETS homologous factor, enhancer of zeste homolog 2 (EZH2), KLF12, NK2 homeobox 2, nuclear receptor co-repressor 2, hairless, hairy and enhancer of split 4, hairy and enhancer of split 7, RUNX1, and v-myb myeloblastosis viral oncogene homolog (avian)-like 1* comprised ∼10% (n = 45) of the upregulated genes (Fig 4). It is important to note that several genes falling under the cell signaling and transcription category are core components of the Wnt and Notch signaling pathway. The increased expression of *EZH2* is interesting as it is a key component of the polycomb repressive complex 2 that trimethylates histone H3 on Lys 27 and represses gene transcription, thereby, functioning as an anti-differentiation factor [24].

Approximately, 5% (n = 22) of the genes represented immune defense and inflammation. Notable genes were *defensin, beta 119, mucin 5B, T cell immunoglobulin mucin 3, interferon induced transmembrane protein 3 (all 4 immune defense stimulators), and inhibitor of kappa light polypeptide gene enhancer in B-cells* (Fig 4).

Important genes regulating cell-cell and cell-matrix interactions (3%, n = 14) included s*idekick homolog 1, CD44, cadherin 8, type 2, cytohesin 1 interacting protein, ninjurin 1, fibronectin leucine rich transmembrane protein 2, and slit homolog 3*.

The remaining genes fell into the following categories: cellular transport (9%, n = 38), cell division/differentiation (8%, n = 31) regulation of cellular cytoskeleton (2%, n = 9), apoptosis (1%, n = 7), translation (2%, n = 7) and DNA replication/repair (2%, n = 4). Among the cell division/differentiation genes *PFTAIRE protein kinase 1 (CDK14)*, a serine threonine protein kinase is conspicuous, as it functions as a cell-cycle regulator of the Wnt signaling pathway during the G2/M phase [25]. *CDK14* has been reported to facilitate the phosphorylation of *low density lipoprotein receptor-related protein 6* at 'Ser-1490′, leading to the activation of the Wnt signaling pathway [25].

Overall, although a surge in the expression of Wnt and Notch signaling genes is evident, the enhanced expression of *EZH2* at viral set point is striking and suggests an important epigenetic mechanism taking center stage with known potential to attenuate cellular differentiation processes while at the same time promoting cellular proliferation.

### Genes encoding proteins associated with tight, adherens junction and desmosome formation are significantly down-regulated suggesting marked impairment of epithelial barrier integrity and absorptive functions by 90 days post SIV infection

The 90DPI time point witnessed the maximum number of down-regulated genes (1605 genes). Most of the down-regulated genes fell into four categories: Metabolism (18%, n = 237), transcription (11%, n = 150), cellular transport (10%, n = 137) and cell signaling (10%, n = 132). The rest of the genes fell into the following categories: cell division/differentiation (5%, n = 68), cell adhesion/migration (4%, n = 56), immune defense/inflammation (3%, n = 39), regulation of cellular cytoskeleton (2%, n = 30), apoptosis (2%, n = 24), translation (1%, n = 17), and DNA replication/repair (1%, n = 12) (Fig 5).

Important down-regulated genes linked to transcription comprised *JMJD3, JARID2, KLF6&10, forkhead box P2&4, pancreatic and duodenal homeobox 1, peroxisome proliferator-activated receptor alpha, TCF7L2 and suppressor of fused*. In addition several cell signaling genes that showed decreased expression comprised *PTK2 protein tyrosine kinase 2, activin A receptor, type IIA, adenomatosis polyposis coli 2, mitogen-activated protein kinase kinase kinase 3, transforming growth factor, beta receptor III, frizzled-related protein, dickkopf homolog 3 and dispatched homolog 1 (Drosophila)*. At least, seven genes previously known to regulate intestinal transport namely, *solute carrier family 15 (oligopeptide transporter), solute carrier family 5 (sodium/glucose cotransporter), solute carrier family 26, member 3, ATP-binding cassette transporter 13, cytochrome b reductase 1 and solute carrier family 4, sodium bicarbonate cotransporter* showed significantly decreased expression.

Although genes that fell into the cell adhesion/migration category only represented 4% of the total several of these have been well characterized and known to regulate epithelial cell function, polarity and homeostasis. Some of the important cell-cell and cell matrix interactions genes that displayed reduced expression included *cadherin 5 type 2, catenin (cadherin-associated protein) alpha 1, claudin 22, desmoglein 2, integrin, alpha 1,3 & 6, laminin, beta 3&4, laminin, gamma 1&2, par-3 partitioning defective 3 homolog,* (*par-3 partitioning defective 3 homolog B*, *par-6 partitioning defective 6 homolog gamma*, *protocadherin 15, desmocollin 2, junction plakoglobin, syndecan1, and cadherin 11, type 2*.

Compared to the 21 DPI time point, several interesting genes associated with cellular polarity (*protein kinase, AMP-activated, gamma 2 non-catalytic subunit*), Notch signaling (*jagged 1*), transcription [*zinc finger E-box binding homeobox 2, RUNX2, SOX2*], immune response/inflammation [*NOD1, dual oxidase 1, and IL-8R B*], transport [*solute carrier family 40 (iron-regulated transporter), member 1, sodium channel, nonvoltage-gated 1, beta*] and cell signaling [*dickkopf homolog 3, ring finger protein 138, EPH receptor A6, ubiquitin specific peptidase 34*] showed decreased expression at the 90DPI. At least, three genes in the cell signaling category, namely, *dickkopf homolog 3* [26], *ring finger protein 138* [27] *and ubiquitin specific peptidase 34* [28] are linked to the Wnt signaling pathway.

**Figure 6:**
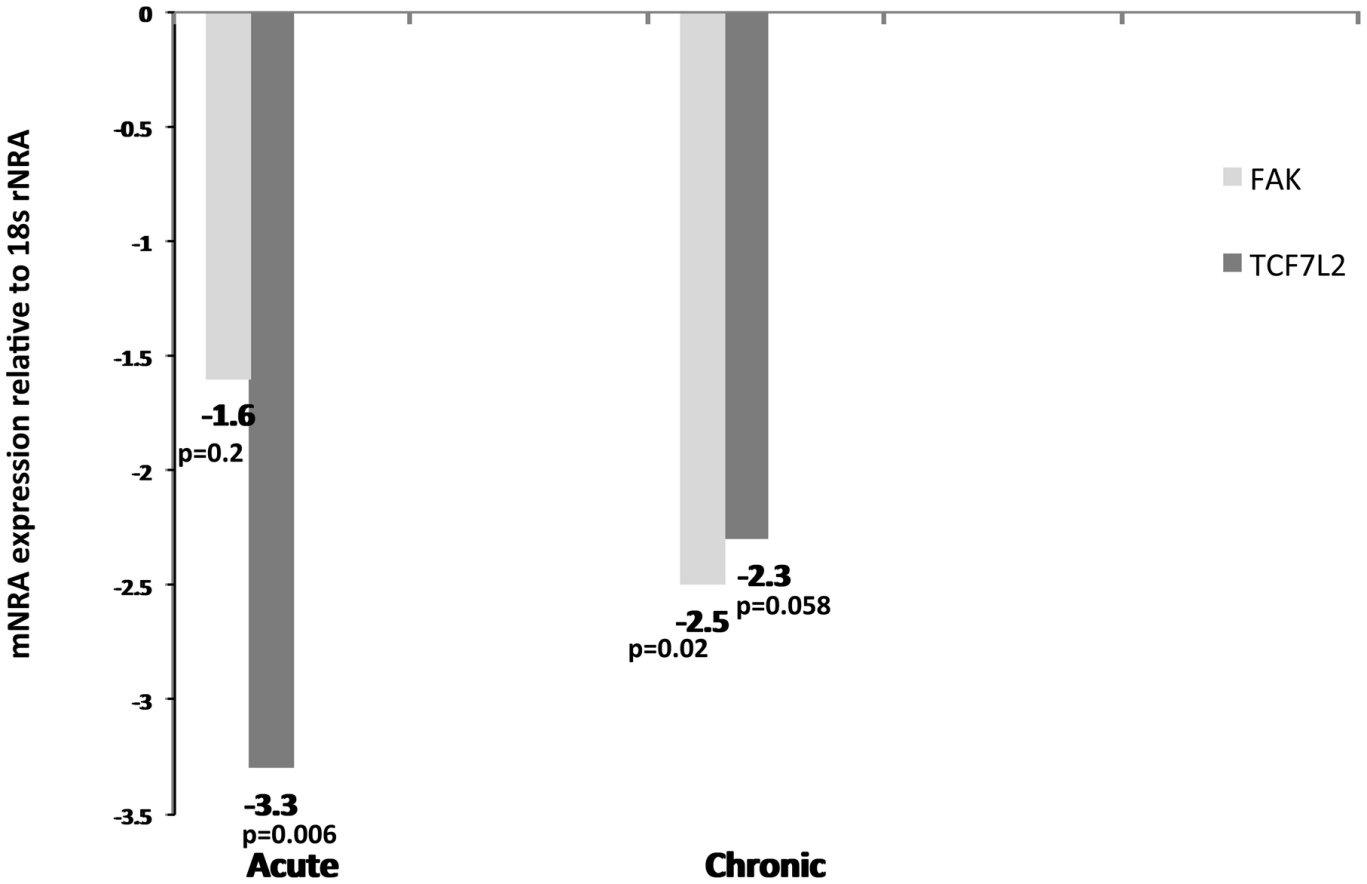
FAK and TCF7L2 expression is significantly decreased in the intestinal epithelium during acute and chronic SIV infection. Relative abundance in gene expression for FAK (light bars) and TCF7L2 (dark bars) in the intestinal epithelial cellular compartment of the jejunum at 21DPI (n = 4) and 6 months (n = 6) post SIV infection detected using quantitative real-time SYBR green two-step RT-PCR. The fold differences in gene expression were calculated as described in *Materials and Methods*. The relative fold increase is shown adjacent to each bar graph. The asterisk (*) indicates statistical significance (*p<0.05*).

We chose *TCF7L2* and *FAK* for further confirmation studies using real-time RT-PCR because of their importance to intestinal epithelial cell proliferation, migration and repair. *TCF7L2* is critical to crypt cell proliferation and its expression is considerably decreased in the ileum of crohn's disease patients [29]–[30]. Further, *TCF7L2* has also been reported to regulate the expression of defensins alpha and beta in paneth cells [29]. Similarly, recent studies show that *FAK* is critical for intestinal epithelial cell proliferation, migration, repair and healing following epithelial injury [31]. As shown in figure 6, quantitative real-time RT-PCR confirmed a statistically significant (*p*<0.05) decrease in the expression of *TCF7L2* at 21DPI and FAK at six months post SIV infection in the intestinal epithelial compartment. Although not statistically significant *TCF7L2* displayed a similar trend (p = 0.58) at six months post SIV infection (Fig 6).

## Discussion

The intestinal epithelium comprises a single layer of differentiated polarized simple columnar cells (enterocytes) that functions in nutrient absorption and at the same time protects the underlying lamina propria cells from the external environment. The enterocytes lining the villi are shed into the lumen every 5 to 7 days to be replaced by new cells that migrate up the villi from the proliferating crypt cell compartment. The proliferation, migration and differentiation of intestinal epithelial cells is tightly controlled by several signaling pathways and their corresponding transcription factors that work in tandem to maintain epithelial homeostasis [32]. Differential gene expression, which is central to these events, is tightly regulated so that proliferation, migration and differentiation occur in a well-controlled and orchestrated manner [32]. However, in GI inflammatory diseases such as inflammatory bowel disease and AIDS the burgeoning pro-inflammatory environment in the lamina propria can have a detrimental effect on the homeostasis of the overlying epithelial cells by altering the epithelial gene expression program. Moreover, in HIV/SIV infection the massive destruction of CD4^+^ T cells can disrupt the lympho-epithelial communication network that can further destabilize the epithelial gene expression program. This is clearly evident in RAG^−^/^−^ mice that show serious defects in epithelial cell differentiation due to the absence of LPLs [33]. Nonetheless, transfer of CD4^+^CD62L^+^CD45Rb^Hi^ and/or CD4^+^CD62L^+^CD45Rb^Lo^ cells into these mice substantially reduced the permeability of the colon [33]. Accordingly, it is reasonable to hypothesize that continual CD4^+^ T cell destruction and subsequent pro-inflammatory cytokine production in the lamina propria can disturb epithelial cell proliferation, migration and differentiation by altering the epithelial cell transcriptome. Consistent with this hypothesis, massive apoptotic loss of epithelial cells has been demonstrated to occur very early in SIV infection [16]–[17]. The response of the intestine, in particular, the crypt cell compartment to this massive early cell death for the purposes of repair and healing is not completely understood and needs detailed investigation. To address this important topic, as a first step, we isolated and compared transcriptional profiles in purified intestinal epithelial cells (a heterogeneous combination of absorptive enterocytes, goblet, enteroendocrine, paneth and crypt cells that contain the intestinal stem cell population) before and at 21 and 90DPI.

Loss of intestinal epithelial cells to apoptosis is a hallmark pathological event reported to occur concurrently with CD4^+^ T cell loss early in HIV/SIV infection [16]. Loss of the lining epithelial cells may offer a partial explanation for the diarrhea experienced by most infected individuals early in the course of the disease [34]. In agreement with the aforementioned clinical findings, in the present study we observed increased expression of pro-apoptotic genes such as *CASP2 and RIPK1 domain containing adaptor with death domain, forkhead box O3 (FOXO3), FOXO1, serine/threonine kinase 17a, serine/theronine kinase 3, tumor necrosis factor receptor superfamily, member 25, WT1-interacting protein, and protein phosphatase 1f*. The accelerated loss of differentiated enterocytes signals the crypts to proliferate and in some severe cases hyperproliferate leading to crypt hyperplasia and villus atrophy, a histopathological change well documented in HIV/SIV infected individuals [35]. The molecular mechanisms underlying crypt hyperplasia remain poorly understood. The Wnt and Notch signaling pathways have been demonstrated to play important roles in regulating morphogenetic and homeostatic events in the intestine [36]. While it was surprising that the expression of genes linked to the Wnt signaling pathway decreased (discussed below), we found elevated expression of *HES6*, a notch target gene and *DLL4*, an important notch ligand at 21DPI. More interestingly, at 90DPI we detected a further escalation in the expression of notch related genes. These included *Notch3*, an important notch ligand, *DTX1 & 3*, positive regulators of notch signaling and *HES4 & 7*, two notch target genes. As disease progresses the burgeoning inflammatory environment in the lamina propria can further exacerbate epithelial cell loss and a sustained surge in notch signaling will be necessary to replenish enterocytes and restore the integrity of the epithelial barrier. The importance of notch ligands (particularly, *DLL1 and DLL4*) is clear from the findings that simultaneous inactivation of *DLL4* and *DLL1* forces progenitor cells to differentiate into the secretory cells (goblet cells) together with the loss of stem cells in the crypt compartment [37]. Accordingly, the increased expression of *DLL4* and *NOTCH3* may provide the stimulus to activate crypt cell proliferation to replace the enterocytes lost to apoptosis early in infection. Similarly, the enrichment of the notch activated target genes *HES4, 6 and 7* mRNA provides strong evidence of active notch signaling in the intestinal epithelium at 21 and 90DPI. These findings fit well with the recent finding that notch signaling is critical for the proliferation of crypt progenitor cells and for their differentiation into absorptive enterocytes [38].

Cyclin D3 is critical for intestinal epithelial cell proliferation [39] and for that reason its increased expression at 21DPI suggests that cells from the crypt cell compartment are entering the cell cycle to divide and replace the lost enterocytes. Proliferating progenitor cells have to gradually migrate up the villi to replace the lost cells with the help of signaling molecules like Ephrin ligands (*Ephrin A and B*) and their receptors (*Eph A and B*) that are known to mediate cell compartmentalization and guide the proliferating cells to migrate before they differentiate [40]. Consistent with their role in guiding cell migration, at least, two Ephrin receptors, namely, *Eph A2* and *Eph B3* showed enhanced expression at 21DPI. Similarly, although notch signaling is required for the differentiation of absorptive enterocytes, the differentiation of goblet and enteroendocrine cells that belong to the secretory class of intestinal epithelial cells are largely dependent on the regulatory functions of the forkhead transcription factor *FOXA2* [41] and *RUNX1* [42] as inactivation of both transcription factors disrupts their differentiation. Increased expression of *FOXA2* and *RUNX1* likely indicates an attempt to induce the proliferating progenitor cells to differentiate into goblet and enteroendocrine cells so that protective mucus and hormone secretory functions of the intestinal epithelium are restored.

Signaling through the Wnt pathway is critical for crypt stem cell proliferation and renewal as deletion of *TCF7L2* function results in complete loss of proliferative cells in the crypt cell compartment of the fetal small intestine [43]. Even though activation of the Wnt pathway has been strongly linked to intestinal crypt cell proliferation we observed decreased expression of several Wnt pathway associated genes such as *Wnt10A, FZD6, SOSTDC1* including the important downstream Wnt transcription factor *TCF7L2* at 21DPI. *Dishevelled 2* was the only Wnt gene that showed increased expression at this time point. At 90DPI several Wnt genes such as *Wnt7B* (Wnt ligand), *DVL1* (stimulator of Wnt signaling), *DAAM1* exhibited increased expression. At the same time inhibitors of Wnt signaling, namely, *DKK1, sFRP, APC* and *TLE1* were down-regulated. The reduced expression of Wnt antagonists and negative regulators at 90DPI is intriguing and may point in the direction of a bid to activate Wnt signaling. Surprisingly, *TCF7L2* showed reduced expression at both 21 and 90DPI. Interestingly, reduction in *TCF7L2* expression has also been reported in other intestinal disorders, particularly, ileal crohn’s disease (CD) or crohn's ileitis but not in colonic CD or ulcerative colitis [29]–[30]. In agreement with these studies we also did not observe decreased *TCF7L2* expression in the colon of SIV-infected macaques (data not shown). Reduction in the *TCF7L2* mRNA expression also resulted in decreased expression of alpha defensins, namely, HD5 and HD6 in paneth cells as the expression of these important antimicrobial proteins are under the direct transcriptional control of *TCF7L2* [29]–[30]. The biological significance of *TCF7L2* downregulation and its underlying mechanisms remain unclear. Also, since *TCF7L2* is an inhibitor of cellular differentiation [44] and a strong promoter of cellular proliferation the decreased expression might be seen as an attempt to drive the newly dividing progenitor cells to differentiate. The significantly reduced expression of *TCF7L2* at 21 and again at 90DPI may suggest growing disturbances in enterocyte differentiation as disease progresses. Alternatively, decreased expression of members of the Wnt signaling pathway may be a protective mechanism to prevent uncontrolled proliferation that would favor intestinal tumorigenesis. Nevertheless, the functional significance of *TCF7L2* downregulation and the underlying mechanisms remain unknown and require future investigation.

Cell adhesion proteins are indispensable for regulating intestinal paracellular permeability (tight junctions, adherens junctions and desmosomes) and also for anchoring polarized epithelial cells to the basement membrane (focal adhesions and hemidesmosomes). This assures that the epithelial cells are correctly oriented to perform the functions of absorption and secretion. Maintaining the integrity of the intestinal epithelium is critical to prevent unwarranted entry of intestinal bacteria and subsequent inflammation. Expression of *FAK, CD164* (both epithelial cell migration and survival), *CD36, v-set and immunoglobulin domain containing 1 (member of junctional adhesion molecule), claudin 22* (both associated with tight junction proteins), *cadherin 5, catenin* (adherens junction), *integrin alpha 6 and integrin beta 1* (receptor for *laminins*) was found to be significantly decreased at 21DPI. As infection continues, at 90DPI, *Angiomotin like-1* (component of tight junctions) and other adherens junction proteins such as *cadherin 11, 23, catenin alpha 1* and *FAK* also displayed significantly decreased expression. In addition, genes encoding components of the hemidesmosomes such as d*esmoglein 2, desmocollin* and *junctional plakoglobin* were also downregulated.

*FAK* plays a critical role in intestinal epithelial survival and healing as mice mutant for *FAK* were highly susceptible to colitis and exhibited increased p53 expression resulting in epithelial cell apoptosis [31]. The reduced expression of *FAK* at 21 and 90DPI may also contribute to continued loss of epithelial cells to apoptosis. Most notably at 90DPI genes encoding laminin proteins, namely, *laminin beta 3&4, laminin gamma 1&2* including their receptors *integrin alpha 1, 3 and 6* showed significantly reduced expression. Laminins belong to a large family of heterotrimeric molecules that localize to the basement membrane of epithelial cells and mediate important functions such as adhesion, proliferation, migration and differentiation. Altered expression of laminin proteins has been previously reported in the small intestinal mucosa of crohn's disease patients [45]. The dysregulated expression of genes encoding cell adhesion molecules suggests that the formation of strong adhesions and cell compartmentalization is not occurring synchronously with epithelial cell proliferation and migration. Consequently, the selective permeability of the epithelial barrier is severely compromised, thus facilitating the unrestricted influx of lumenal bacteria and their products into the systemic circulation, thus promoting localized and systemic inflammation/immune activation.

Unlike the classical cell adhesion molecules, *sidekick homolog 1,* an interesting cell adhesion molecule associated with HIV associated nephropathy, was found to be significantly upregulated at 90DPI. *SDK1* expression is considerably elevated in kidney, particularly, in the podocytes of HIV infected individuals. *SDK1* causes dedifferentiation of podocytes and induces their uncontrolled proliferation leading to glomerulosclerosis and nephropathy [46]. The role of *SDK1*, especially its increased expression, in the intestinal epithelium is unclear and whether it induces a similar dedifferentiation response in the intestinal epithelium requires future investigation.

In addition to cell adhesion molecules, genes linked to the establishment of epithelial cell polarity also showed significantly decreased expression. These encompassed *lethal* (21DPI), *PARD3B homolog B and C* (*PAR-3B*) and *PARD6 homolog gamma (PAR-6G)* (90DPI). *PARD3B* (partitioning-defective) is a scaffold-like PDZ (postsynaptic density-95/discs large/zonula occludens-1) domain-containing protein that forms a heterotrimeric complex with *PAR-6* and atypical PKC [47]. The complex has been localized to tight junctions of epithelial cells and reported to contribute to the formation of functional tight junctions [47]. Further the expression of *PARD3B* is markedly altered in intestinal inflammatory diseases leading to defects in epithelial tight junctions [48]. This suggests that *PARD3B/PARD6BG* complexes not only are critical to the formation of epithelial tight junctions but also to the establishment of apical and basal surfaces.

The cell adhesion molecules, *Ezrin,* also known as *villin-2* also displayed decreased expression at 90DPI. *Ezrin* has been reported to play an indispensable role in organizing the apical domain of polarized epithelial cells by assembling multiprotein complexes that stabilize the membrane-cytoskeleton interface [49]. Overall, the reduced expression of genes encoding cell adhesion molecules and the establishment of epithelial cell polarity suggests defects in maturation/differentiation of enterocytes. This may be due to the fact that the epithelial repair and healing mechanisms are outpaced by several factors such as the lack of epitheliotrophic factors originating from CD4^+^ T cells, increased production of proinflammatory cytokines by lamina propria immune cells and the expression of new chromatin modifying proteins (see below).

While the decreased expression of critical cell adhesion molecules, *FAK, PDX1* and the downstream Wnt transcription factor *TCF7L2* represent major findings of this study, the underlying mechanisms remain unclear. Nevertheless, the increased expression of *EZH1* at 21 DPI and *EZH2* at 90DPI together with a concomitant decrease in the expression of *JMJD3* at 90DPI suggests that an epigenetic mechanism involving histone modifications may play a role in transcriptional silencing. *EZH2* is a histone lysine methyltransferase known to trimethylate “Lys-27” on histone H3 [24]. It is a component of the polycomb-repressive complex-2 and functions by transcriptionally silencing genes that regulate developmental programs in stem or progenitor cells including cancer cells [24]. By doing this, *EZH1* and *EZH2* helps maintain stem cell identity by inhibiting cellular differentiation programs [24]. Similarly, *EZH2* has been reported to boost tumor growth by targeting signaling molecules that promote cellular differentiation and at the same time stimulating cell cycle progression [50]. The likelihood of *EZH2* playing a role is further strengthened by the decreased expression of *JMJD3* (Jumonji containing domain 3) histone demethylase that specifically demethylates trimethylated and dimethylated 'Lys-27′ of histone H3, a process that reverses the changes created by *EZH2* thereby enabling transcriptional activation [51]. The bimodal expression of *EZH2* and *JMJD3* may also explain the marked increase in the number of transcripts that displayed reduced expression at 90DPI. Finally and more importantly, these significantly new findings on polycomb mediated transcriptional regulation would not have been possible had we used intact intestinal segments as it would have been virtually impossible to determine the cellular origins of these critical chromatin modifying enzymes.

The findings from the present study provide an in depth analysis of the molecular changes at the level of transcription occurring exclusively in the intestinal epithelium immediately following the CD4^+^ T cell loss until the establishment of viral set point. These findings, to our knowledge for the first time provide valuable information on the altered regulation of *Wnt* and *Notch* signaling pathways and cell adhesion molecules in the intestinal epithelium following SIV infection. Further, the unambiguous assignment of the unique transcriptional signatures to the intestinal epithelial compartment would not have been possible had we used intact intestinal segments. Future work is necessary to understand the mechanisms underlying altered expression of several important genes such as *TCF7L2*, and *sidekick homolog 1*. While considerable effort has been devoted to studying tight junction and adherens junction proteins the findings from the present study provide a valuable reminder that apart from cell-cell, cell-matrix adhesions mediated by hemidesmosomes (laminins) require more scrutiny in the future. Additional studies involving *in situ* hybridization/immunofluorescence, western blotting,. etc are required to validate the differentially expressed genes as well as further investigate the role played by individual signaling pathways in regulating epithelial cell proliferation, differentiation and function. Similar high throughput studies incorporating the intraepithelial lymphocytes and fibrovascular stroma in the immediate future will add greater insight into the molecular mechanisms underlying GI dysfunction.

## Materials and Methods

### Ethics statement

All experiments using rhesus macaques were approved by the Tulane Institutional Animal Care and Use Committee (Protocol 3267-B00). The Tulane National Primate Research Center (TNPRC) is an Association for Assessment and Accreditation of Laboratory Animal Care International accredited facility (AAALAC #000594). The NIH Office of Laboratory Animal Welfare assurance number for the TNPRC is A3071-01. All clinical procedures, including administration of anesthesia and analgesics, were carried out under the direction of a laboratory animal veterinarian. Animals were anesthetized with ketamine hydrochloride for blood collection procedures. Intestinal resections were performed by laboratory animal veterinarians. Animals were pre-anesthetized with ketamine hydrochloride, acepromazine, and glycopyrolate, intubated and maintained on a mixture of isoflurane and oxygen. Buprenorphine was given intra-operatively and post-operatively for analgesia. All possible measures are taken to minimize discomfort of all the animals used in this study. Tulane University complies with NIH policy on animal welfare, the Animal Welfare Act, and all other applicable federal, state and local laws.

### Animals and Tissue Collection

Serial resection biopsies (∼6–8 cm long) of jejunum were collected from three Indian-origin rhesus macaques prior to infection and 21 and 90DPI with SIVmac251 for microarray studies. All animals were infected intravenously with 100TCID_50_ of SIVmac251 grown on CMEX174 cells. Sequential Intestinal resection surgeries are routinely performed at the TNPRC on rhesus macaques without any detrimental effects on the animal's health. The resections in the present study were done months apart giving sufficient time for healing and repair processes to be complete. For quantitative RT-PCR confirmation studies, jejunal tissues from ten additional SIV-infected macaques (four animals at 21DPI and six at 6 months PI) and six uninfected control macaques were processed as described below.

### Cell isolation from Intestinal resection segments

In order to determine the impact of high viral replication and massive CD4^+^ T cell loss on the intestinal mucosa we conducted a longitudinal study to assess genome wide changes in gene expression profiles during SIV infection using Affymetrix (Santa Clara, CA) rhesus macaque arrays that contain about 54,675 capture probes. To minimize information loss and to make the starting material less complex we separated the intestinal epithelial cells from the underlying LPLs and fibrovascular stroma. Finally, the intra-epithelial cells (IELs) were separated from the epithelial cells and changes in gene expression were analyzed in all 4 compartments separately. In order to successfully separate all 4 tissue compartments and ensure the availability of sufficient starting material we obtained intestinal resection segments (6–8 cm long) from the jejunum instead of pinch biopsies. We recently reported changes in transcriptional profiles in the lamina propria cell compartment following SIV infection [12]. In the present communication we have focused on the changes occurring in the jejunal epithelium at 21 and 9DPI. Comparisons in gene expression were made to resection segments collected from the same animal 6 weeks prior to SIV infection.

Briefly, surgical resection segments (6–8 cm long) for mRNA profiling studies were first incubated with vigorous shaking in Ca^++^Mg^++^ free-HBSS containing 1 mM EDTA for two 30-min incubations at 37°C to separate the intestinal epithelial cells [2], [52]. Following incubation, the epithelial cells in the supernatant were harvested by centrifugation at 500 g for 10 min followed by subjecting the cells to percoll density gradient centrifugation to separate IELs [2], [52]. This protocol has been demonstrated to yield epithelial cells with >85% purity with minimal contamination with IELs [17].

### Phenotyping blood and tissue mononuclear cells

Peripheral blood mononuclear cells (PBMCs) were isolated and processed as previously described [52]. PBMCs were collected by centrifugation over lymphocyte separation media. Cells (PBMCs and LPLs) were adjusted to a concentration of 10^7^/ml and 100 μl aliquots (10^6^ cells) were stained with appropriately diluted, directly-conjugated monoclonal antibodies to CD45RA fluorescein isothiocyanate (FITC), CCR5 phycoeryrthrin (PE), CD8-peridinin chlorophyll A protein (PerCP) and CD4-allophycocyanin (APC) (all from BD Biosciences Pharmingen San Diego, CA). Samples were stained for 30 min in the dark at 4°C, fixed in 2% paraformaldehyde, and stored in the dark at 4°C overnight for acquisition the next day. Samples were acquired on a LSR II flow cytometry equipment (BD Biosciences) and analyzed with Flow Jo software (Treestar Inc, Ashland, OR). Samples were first gated on lymphocytes by forward and side scatter plots and then through CD3+ lymphocytes, and finally CD4^+^ or CD8^+^ T cells. Changes in CD45RA+/CCR5- populations at the 21 and 90DPI timepoints were analyzed using the wilcoxon matched-pairs signed rank test.

### Microarray Hybridization and Statistical Analysis

Microarray-based profiling of genome wide changes in mRNA expression in epithelial samples was performed using Affymetrix rhesus monkey GeneChips (U133A 2.0). RNA was isolated from the three epithelial samples derived from intestinal resection seqments collected at 6 weeks before and at 21 and 90d post-SIV infection. Total RNA was used to synthesize double-stranded cDNA (Superscript Choice System; Life Technologies Bethesda Research Laboratories). The resulting cDNA was purified and used for *in vitro* transcription to produce biotin-labeled cRNA (BioArray HighYield RNA Transcription Labeling kit; Enzo Diagnostics). The biotinylated cRNA was cleaned (RNAeasy Mini kit; Qiagen), fragmented, and hybridized on GeneChips containing 54,675 probes sets, using standard protocols at a commercial GeneChip core facility. Following three washes, individual GeneChips were stained with streptavidin-phycoerythrin (Molecular Probes), amplified using biotinylated anti-streptavidin (Vector Laboratories), and scanned for fluorescence (GeneArray Scanner; Hewlett Packard) measurement on a Microarray Suite 5.0 software (MAS 5.0; Affymetrix).

For data analysis, the Affymetrix CEL files (containing scanned images, together with absolute calls for each gene) were transferred to the S+ statistical module within the Spotfire DecisionSite for Microarray Analysis (TIBCO-Spotfire) program. Chips were normalized using the Robust Multichip Analysis (RMA) method, to stabilize MvA plots. This step was essential to eliminate any intensity-specific bias in probe-level data and to produce a matrix comprising of normally distributed data. Expression indices were reported as log (base 2) of change in gene-expression at either 21 or 90DPI time-points relative to a common pre-infection RNA (obtained from all 3 animals) as a reference or baseline. Probe sets whose targets were not detected were removed from the data matrix. A Student's t test was then performed to identify genes expressed in a statistically significant manner (*P<0.05*). A fold change cutoff of ≥1.5-fold in all three macaques at 21 and 90DPI time points was then applied, so as to only consider genes whose expression was perturbed in magnitude and in a statistically significant manner. All genes listed in Tables S1 and S2 including the pie charts (Figure 2, 3, 4, 5) were found to be differentially expressed above or below the cut-off in all three animals.

Gene ontology/annotation analysis was performed using the DAVID (Database for Annotation, Visualization and Integrated Discovery) Bioinformatics Functional Annotation tool (http://david.abcc.ncifcrf.gov) [13]–[14] and GeneCards® (http://www.genecards.org/) [15] on all differentially (Up and Down) expressed transcripts.

### Quantitative Real-Time SYBR Green two-Step RT-PCR

Gene expression for *FAK* and *TCF7L2* in the jejunal epithelial compartment of ten SIV infected macaques (four animals at 21DPI and six at 6 months PI) was further evaluated by Quantitative Real-Time SYBR Green Two-Step RT-PCR assay (QRT-PCR) (ABI, Foster City, CA). Total RNA was extracted using the miRNeasy kit (Qiagen Inc, Valencia, CA) and reverse transcribed using the SuperScript. III First-Strand Synthesis System for RT-PCR kit following the manufacturer’s protocol. Each QRT-PCR reaction (20 μl) contained the following: 2X Power SYBR Green Master Mix without uracil-N-glycosylase (10 μl), target forward and reverse primer (200 nM) and cDNA (4 μl). Forward and reverse primer sequence for *FAK*, *TCF7L2* and *18* *s rRNA* is shown in Table 4. The PCR amplification was carried out in the ABI 7900 HT Fast PCR System (Applied Biosystems, Foster City, CA). Thermal cycling conditions were 95°C for 10 minutes followed by 40 repetitive cycles of 95°C for 15 sec, 60°C for 1 min. As a normalization control for RNA loading, parallel reactions in the same multiwell plate were performed using 18 s rRNA (18 s).

**Table 4:** Primer sequences used for real time Power SYBR Green Two-step RT-PCR.

Gene Name	Primer sequence	Product size (bp)	Primer concentration
FAK	For- 5′- GTGAGGTGCAGGACAAGTATGAGT-3′	76	200 nM
	Rev- 5′-GGCAAGTAGCGGATTTGAAGGTCA-3′		
TCF7L2	For-5′- GCTGAATGATTTACTGGATTTCAGTGCGAT-3	86	200 nM
	Rev-5′-GTCCACTTGCCAAAGAAGTTGGTC-3′		
18srRNA	For-5′- GCTACCACATCCAAGGAAGGCA-3′	100	200nM
	Rev-5′- AGGGCCTCGAAAGAGTCCTGTATT-3′		

Quantification of gene amplification following RT-PCR was made by determining the threshold cycle (C_T_) number for SYBR Green fluorescence within the geometric region of the semi-log plot generated during PCR. Within this region of the amplification curve, each difference of one cycle is equivalent to a doubling of the amplified product of the PCR. The relative quantification of target gene expression across treatments was evaluated using the comparative C_T_ method. The ΔC_T_ value was determined by subtracting the 18s C_T_ value for each sample from the target C_T_ value of that sample. Calculation of ΔΔC_T_ involved using the highest sample ΔC_T_ value (i.e., sample with the lowest target expression) as an arbitrary constant to subtract from all other ΔC_T_ sample values. Fold changes in the relative gene expression of target was determined by evaluating the expression, 2^−ΔΔCT^. The data was analyzed using RealTime StatMiner™ package, a bioinformatics software developed by integromics, on Spotfire DecisionSite.

## Supporting Information

Figure S1 Jejunum viral loads in all three animals at necropsy (90 days post infection).
(TIF)

Table S1 The full list of differentially expressed genes showing statistical significance at 21 days after SIV infection with their affymetrix IDs, gene and functional annotation. Tab 1: Up in 21d PI, Tab 2: Down in 21d PI, Tab 3: Up in 21d compared to 90d PI.
(XLSX)

Table S2 The full list of differentially expressed genes showing statistical significance at 90 days after infection with their affymetrix IDs, gene and functional annotation. Tab 1: Up in 90d PI, Tab 2: Down in 90d PI, Tab 3: Up in 90d compared to 21d PI.
(XLSX)
