# Supplementary figures and images for: Focused Examination of the Intestinal Epithelium Reveals Transcriptional Signatures Consistent with Disturbances in Enterocyte Maturation and Differentiation during the Course of SIV Infection
Source: PLoS One. 2013 Apr 9;8(4):e60122. doi: 10.1371/journal.pone.0060122 (PMC3621888; doi:10.1371/journal.pone.0060122)

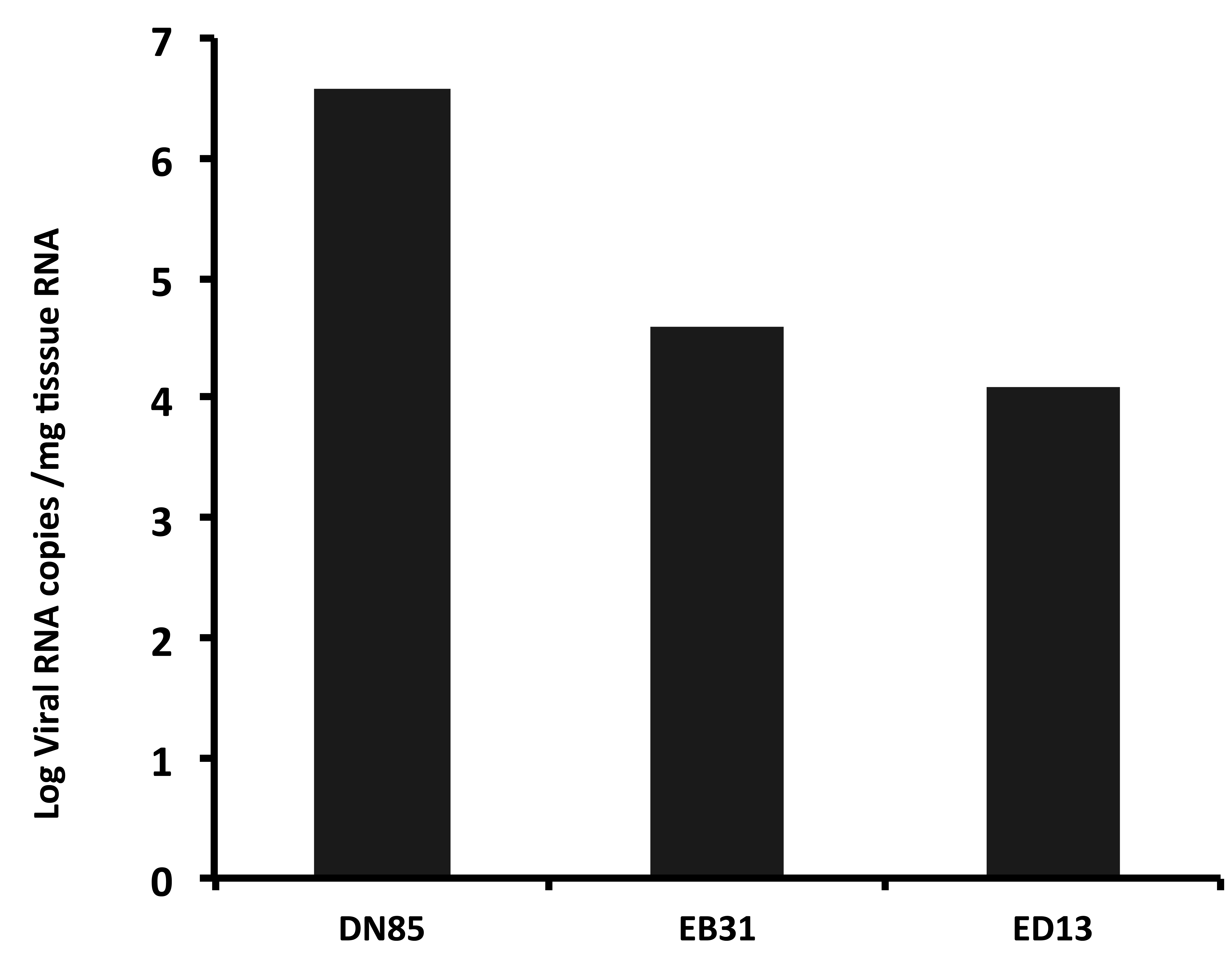

Supplement: Figure S1 — Jejunum viral loads in all three animals at necropsy (90 days post infection). (TIF) [file pone.0060122.s001.tif]
